# The Twitter parliamentarian database: Analyzing Twitter politics across 26 countries

**DOI:** 10.1371/journal.pone.0237073

**Published:** 2020-09-16

**Authors:** Livia van Vliet, Petter Törnberg, Justus Uitermark

**Affiliations:** 1 Department of Sociology, University of Amsterdam, Amsterdam, Netherlands; 2 Department of Space, Earth and Environment, Chalmers University of Technology, Gothenburg, Sweden; 3 Amsterdam Institute for Social Science Research, University of Amsterdam, Amsterdam, Netherlands; Universitat de Barcelona, SPAIN

## Abstract

This article introduces the Twitter Parliamentarian Database (TPD), a multi-source and manually validated database of parliamentarians on Twitter. The TPD includes parliamentarians from all European Free Trade Association countries where over 45% of parliamentarians are on Twitter as well as a selection of English-speaking countries. The database is designed to move beyond the one-off nature of most Twitter-based research and in the direction of systematic and rigorous comparative and transnational analysis. The TPD incorporates, in addition to data collected through Twitter’s streaming API and governmental websites, data from the Manifesto Project Database; the Electoral System Design Database; the ParlGov database; and the Chapel Hill Expert Survey. By compiling these different data sources it becomes possible to compare different countries, political parties, political party families, and different kinds of democracies. To illustrate the opportunities for comparative and transnational analysis that the TPD opens up, we ask: What are the differences between countries in parliamentarian Twitter interactions? How do political parties differ in their use of hashtags and what is their common ground? What is the structure of interaction between parliamentarians in the transnational debate? Alongside some interesting similarities, we find striking cross-party and particularly cross-national differences in how parliamentarians engage in politics on the social media platform.

## 1 Introduction

While many authors have argued that social media data have the potential to revolutionize social science research [1, 2], scholars are just beginning to discover how they can use this new source of data to carefully design social research [3]. To make substantive and rigorous contributions to current debates in the social sciences, further steps need to be taken in terms of developing standards for data collection, preparation and analysis [3, 4]. This is particularly true for Twitter. Since this platform has afforded researchers comparatively broad access, a huge number of studies have drawn on Twitter’s data to study a wide range of social processes. While the excitement about the affordances of Twitter is understandable, few studies have begun to address the formidable challenge of systematically collecting valid and representative data [4–6].

We contribute to this endeavor by presenting a database of parliamentarians on Twitter. The Twitter Parliamentarian Database (TPD) contains all the tweets of members of 27 parliaments (26 national parliaments and the European parliament). The database has been painstakingly and systematically validated to address issues of reliability and validity characteristic of much of the existing research on Twitter politician communication. To address issues of data availability, the TPD incorporates, in addition to data collected through Twitter’s streaming API and governmental websites, data from the Manifesto Project Database on the parliamentarians’ political parties [7]; the Electoral System Design Database on the countries’ electoral and legislative systems [8]; the Chapel Hill Expert Survey on party positions on specific issues [9]; and the ParlGov database on political parties, elections and cabinets [10].

In this paper, we carry out a tentative analysis using the data in order to demonstrate the potential of the database. While the TPD allows for a wide range of analyses, we focus our demonstration on comparative and transnational research, as the database fills a considerable research gap in these fields resulting from the lack of large-scale data sources. To illustrate the capacity of the database to answer questions pertaining to this research, we carry out three illustrative and exploratory studies. (1) We look at parliamentarian’s Twitter use across nations, with a focus on coalitions and divisions through who retweets whom, asking: *What are the differences between countries in parliamentarian Twitter interactions?* (2) We look at differences between how different parties label political issues, asking: *how do political parties differ in their use of hashtags and what is their common ground?* (3) We study the structure of mentions between parliamentarians internationally, asking: *what is the structure of interaction between parliamentarians in the transnational debate?* We do not here aim for definitive answers, but rather use the analyses to illustrate the affordances of the database in relation to research questions that were previously difficult to address. Through including a variety of analyses we demonstrate the different functionalities and areas of research that the TPD is able to touch upon, especially analyses of communication patterns between and within a large number of countries, as well as the content of the communication. While the affordances of Twitter are the same across all the countries, we find cross-party and particularly cross-national differences in how parliamentarians engage in politics on the social media platform.

## 2 Limitations and possibilities of research on Twitter politics

### 2.1 Limitations

Twitter is not only the social media of choice for politicians, but also the social media data source *non plus ultra* for social scientific research [11]. There has been a veritable explosion of research on Twitter in general and Twitter politics in particular (e.g. [12–17]). The Web of Knowledge database contained 10,653 articles with “Twitter” as a key word (as per 31st October 2019), with 4,112 papers produced between January 1, 2018—October 31, 2019. The combination of key words “Twitter” and “politics” finds 640 articles, with 279 published in 2018-2019—roughly one article about Twitter and politics every 3 days. Despite this large and growing body of research, there remain a number of fundamental issues with carrying out research on Twitter data, in particular in terms of *delineation, sampling*, and *validation*.

It has long been established that the *delineation of the population* are poorly addressed in many studies examining politics on Twitter [6, 18]. Researchers often assume that the relevant population consists of Twitter users who index their tweets with specific hashtags. However, there are some serious issues to consider. One is that Twitter users participating in debates do not necessarily use hashtags associated with a particular issue and Twitter users who do use those hashtags do not always participate in the discussion, raising difficult questions of how to decide whether a message is relevant or merely “noise” [19]. Moreover, we know that Twitter users with different political positions tend to use different hashtags [20]. While careful curation of hashtags can attenuate these problems, it cannot solve them; when tweets and users are selected through hashtags, the delineation of the population remains arbitrary to a (generally unknown) extent. When the population is arbitrarily defined, all subsequent analyses can provide evocative results at best. There are however notable attempts at tackling this issue, for instance, Bruns et al. [21] have mapped follower/followee relations to get a more relevant sample of the Australian Twittersphere.

A second issue is associated with the common use of Twitter’s free Streaming API for *sampling* tweets containing keywords. While research into the Twitter Streaming API is relatively sparse and may not be up-to-date due to constant API changes, the research that has been carried out has had troubling implications for the standard approaches to gathering Twitter data, in particular the use of search word or hashtag queries. For instance, when the free streaming API was compared with paid access to the Firehose API (which reportedly gathers 100% of all tweets), the sample became less representative as the number of parameters requested increased [22–24]. The issue here is not just that samples of selected keywords are not representative but that we do not know how samples are drawn and what their biases are. Other methods of sampling have been attempted by researchers through focusing on a core group of individuals, wherein all tweets can be gathered [16]. However, no attempts to standardize sampling at the scale of the TPD have yet been made.

A third problem with the way that Twitter data is used in research is linked to the lack of contextual or background information. Twitter data tend to be difficult to connect to other data sources, meaning that little is known on the identities and political leanings of the users. Consequently, *validation* is difficult. However, contextual information is essential if we want to compare between or within different political groups. In response to this issue, researchers often attempt to infer political viewpoints from behaviors such as a follower or friend network, and hashtag use [25–28]. For instance, if people use, or are listed as, #tcot (i.e., top conservatives on Twitter), it is assumed that they are conservatives. In this case, such a strategy may result in many false positives, but will also only identify a small subset of conservative users. Also, the political viewpoints garnered through these methods may not be representative of the wide variety of political attitudes that exist, where someone may share conservative beliefs in some points, but not in others [29, 30]. The issue of misidentifying users’ political leanings is aggravated by the presence of sock puppets, trolls, and bots (e.g. [19]). Moreover, inferring political views from behavior brings researchers into a legal and ethical grey zone since Twitter regulations forbid the algorithmic identification of users’ political viewpoints. In response to these challenges, researchers have developed more sophisticated methods (e.g. [30–33]) to identify political viewpoints, including linking Twitter data with survey data [30, 34]. In sum, there have been some interesting ways that researchers have tried to circumvent the methodological and technical challenges posed in classifying political viewpoints. We offer a different solution by sampling parliamentarians, wherein party affiliations are known, as well as draw upon existing databases for supplementary information.

### 2.2 Possibilities

If we want to exploit the opportunities for comparative and transnational research into politics that Twitter offers, the issues of delineation, sampling and validation have to be resolved. One way forward, which is the method employed by the database at hand, is to not define populations according to the content of their tweets but to construct a panel of Twitter users whose tweets are collected over time (e.g. [35, 36]). This approach has been taken in a number of studies that have focused on accounts of the United States’ Congress, for which reasonably reliable lists are available on Twitter (see, e.g. [37–39]). Some researchers have begun to study Twitter parliamentarians outside of the United States but often consider only one or at most two countries (e.g. [16, 40, 41]), often in the specific context of election campaigns [12, 42–45]. Thus, while there is considerable research focusing on Twitter use by parliamentarians, to our knowledge, there is no research that includes a large number of countries or that connects to existing databases on parties and parliaments. Hence, the database provides new opportunities for comparative research between multiple countries by making data available that has been gathered over several years from a clearly delineated population.

Focusing on parliamentarians limits the scope of research, but the advantage is that the TPD attempts to resolve several important issues regarding the delineation of the population and sampling in a way that is straightforward, transparent, and verifiable. An additional advantage is that parliamentarians are public figures, which significantly reduces ethical issues regarding privacy protection and increases possibilities to match data obtained from Twitter with data from other sources. In an effort to capitalize on these advantages, the database presented in this article extends earlier work by: including a much greater number of countries; complementing Twitter data with data drawn from other sources; and developing elaborate procedures to maintain data accuracy. The TPD data enable a broad range of Twitter research (e.g. [12, 46–50]). While previous research focused on a limited number of cases or had to rely on convenience samples (e.g. [25, 31, 49]), the TPD allows for studying these topics at scale and in comparative and transnational perspective. As such, we present a variety of analyses to demonstrate the different functionalities of the TPD.

Several research domains can reached by the TPD through linking it to other databases, such as; the Electoral System Design Database (ESDD), the Manifesto Project, the Chapel Hill Expert Survey (CHES) and ParlGov—meaning that the TPD can extend existing comparative work to Twitter, to examine differences in for example the ways parliamentarians express themselves and engage with one another, within nations, within parties as well as transnationally. To illustrate the TPD’s potential, we focus on three domains of comparative research as a demonstration of how the database could begin to answer questions in these areas.

We use several methodologies to illustrate the TPD’s potential for cross-national and comparative research. First, we can use the TPD to investigate national differences in the way parliamentarians use Twitter. Political science literature has a long-standing tradition of comparing political culture and political debate among various nations [51–55], showing important differences across countries and different types of electoral and political systems. We use this literature as a way both of showing how the structure of national retweet networks can shed new light on Lijphart’s classic ideas [51] on the relationship between democracy types and national political communication. Second, the TPD allows for comparison of hashtag use between different parties. Twitter provides textual data that captures the way parliamentarians express themselves and frame political issues. Hence, the TPD constitutes a powerful tool for studying subsets of political party discourse. Despite the apparent potential, there has been limited research on content of parliamentarians’ tweets, and none that are on a large international scale [56]. Therefore, the TPD offers the opportunity to contribute to comparative work on discursive conflicts [57, 58] that so far had to rely on newspaper data that cover only a very small portion of political claims [59]. A third domain in which the TPD can be used is transnational communication. Since social media are often regarded as conduits for breaking geographic boundaries [60], there is a need for systematic analysis of communication flows between countries. Since the TPD not only records interactions within but also between countries, it allows for the examination of the prevalence, nature, sources, and topics of international communication networks on Twitter.

### 2.3 Limitations

The limitations of exclusively following parliamentarians on Twitter should be acknowledged. First, as mentioned, the focus on parliamentarians limits the scope of the research, since parliamentarian tweets only constitute a subset of political discussions on Twitter. This limits usefulness in relation to, for instance, campaigning research, as the TPD only gathers data on parliamentarians who are already in office. It should also be noted that in some cases, parliamentarians may choose not to individually interact with constituents but rather present themselves through the party account. Further, the timeliness of updating the database following elections is largely dependent on the updating of official government websites, which may not occur in some countries until coalitions are formed. In rare cases, this may be several months. While the TPD has a limited scope outside of incumbent parliamentarian communication, it may serve as a starting point for questions about other political debates, by using various techniques to expand from parliamentarian users to other parts of Twitter.

## 3 Data collection and database design

In selecting countries, we aim to contribute to the large and growing body of comparative and transnational analysis. We included all member states and candidate member states of the European Free Trade Association (EFTA) where over 45% of parliamentarians are on Twitter [61]. In addition, we included a number majority English speaking countries because they allow for the application of English text analysis tools and have different political systems than most EU and EFTA countries, thus contributing to variation in the dataset. The countries included are Austria, Belgium, France, Denmark, Spain, Finland, Germany, Greece, Italy, Malta, Poland, Netherlands, United Kingdom, Ireland, Sweden, New Zealand, Turkey, United States, Canada, Australia, Iceland, Norway, Switzerland, Luxembourg, Latvia and Slovenia. In addition, the database includes the European Parliament. A full list of the countries and the proportion of their parliamentarians that use Twitter can be found in the S1 Table.

To identify the *parliamentarians* in the TPD, we consulted official government websites and retrieved all the identities of incumbent members. From these websites, we also collected the parliamentarians’ party affiliations and when available, data on regions and constituencies. When the official government websites did not provide the party information for parliamentarians (as was the case for Germany, France, Finland and Spain), we used Google searches to establish party affiliation. For those websites where English was not available, it was double checked with translation programs that the list we obtained from these websites is the most current and up-to-date list of incumbent members. Identifying parliamentarians on Twitter is occasionally challenging due to e.g. common names and parody accounts, so we followed a protocol to identify and verify Twitter accounts, which involved comparing pictures with those on the government website, examining the number of followers, scrutinizing the lists of followers, and reading the content of the tweets. If the tweets were not in English, they were translated through Google Translate.

Due to the data protection regulation, we only include parliamentarians that are in public service during the time of data collection. Hence, persons who are not currently in parliament but are campaigning to be elected are excluded, as are those who served in one legislative period but were not re-elected in the next. If an account was set to private, it was not included. Inactive accounts, defined as accounts that have not made public tweets after 2014, were also excluded. For the year of 2018, the Twitter Parliamentarian Database captured 6,281,684 tweets from 6,437 parliamentarians active on Twitter out of a possible 8,098, meaning that 79.6% of parliamentarians had an active Twitter account.

The tweets of the parliamentarians are collected using Twitter’s streaming API, including their mentions, retweets, and hashtags. As mentioned with regards to prior research, the streaming API can encounter certain limitations due to its rate limit, which, when used to follow a certain query (e.g. a popular hashtag that may be used 1,000s of times per second), may be reached much faster than following a certain user, who may only tweet a couple of times per day. Hence, following certain user (in this case, a parliamentarian) results in the streaming API gathering almost all tweets of that user as the amount of tweets per minute rarely exceeds the rate limit imposed by the API.

The database is updated following elections. Therefore there may be several electoral periods for some countries, if they had an election during the time of data collection. For example, if persons A and B are elected in 2015, and there is an election in 2018, where person B is reelected but person A is not, there will be data for person B across both periods, but only for person A until the election in 2018. Data collection for a parliamentarian starts from after they are elected, hence their tweet ids before the election date are not included. The Twitter accounts of members of new parliaments are checked for several months following elections, since we discovered that it takes time for new parliamentarians to find their footing on Twitter, and that some parliamentarians may later create new accounts for their parliamentary service, separate from their personal or campaign accounts. The database includes data from as early as May 2017 for some countries, and has been continually updated since, capturing many interesting political events such as the span of Donald Trump’s presidency, the Catalonia referendum, the Brexit debate. The user ids from the database can be used to retrieve older tweets from parliamentarians across the electoral periods where members were collected, or be used to gather further data. Hence, any researcher can use the Twitter ids currently provided in the TPD as a starting point for their own research to update a country of interest following an election.

We conducted cross-validation to confirm the coverage and validity of the collected users and tweets. For user accounts, only limited validation could be carried out, due to the limited availability of databases against which to cross validate. We however compared the TPD ids for members of the US congress against the *115th U.S. Congress Tweet Ids* dataset [62], finding that 93% of the ids in the member list matched. The ids that did not match in either data set were found to be missing accounts that have since been deleted. This could be due to changing accounts during time in office, which can happen as some parliamentarians have campaigning accounts. Moreover, the method of gathering accounts for the 115th U.S. Congress Tweet Ids dataset was different to the TPD, where the former mostly retrieved Twitter accounts from the congress website, which may not differentiate between campaigning and service accounts, rather than manual research which was used for the TPD. To validate the coverage of tweets, we used a random sample of 50 current parliamentarians in the TPD, and retrieved tweets from their timelines occuring between March 1, 2020 and May 1, 2020, using Twitter’s REST API. We confirmed that 98% of the tweet ids that were retrieved from the timelines were found in the database. The 2% that were not found are thought to be due to server downtime.

**Fig 1:**
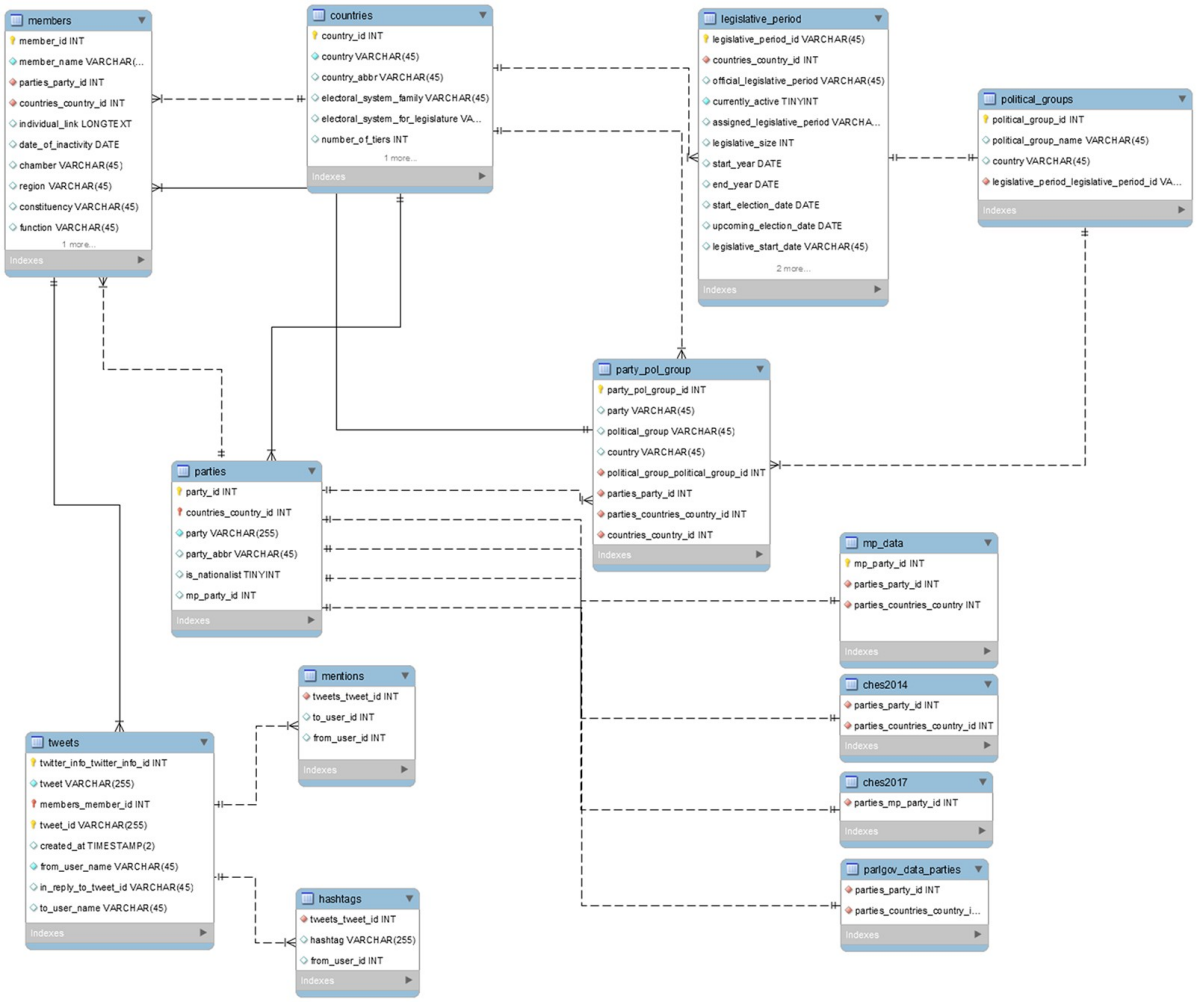
Entity relationships in the TPD. Fig 1 shows a simplified version of the entity relationships between tables in the database, the foreign keys, and their data types. (Note that not all data columns are included in the diagram for the sake of brevity).

Moreover, the database can be connected to the ESDD, the Manifesto Project, CHES and Parlgov, which enables research questions beyond countries, parliamentarians and their parties. The Manifesto Project, CHES and ParlGov can be used with the TPD in various ways. For instance, the ESDD can be used in combination with Twitter data to determine relationships between electoral systems and online politician communication, where additional variables like electoral size, number of tiers and legislative system are also available [8]. The manifesto project provides “parties’ policy positions derived from a content analysis of parties’ electoral manifestos.” [7] ParlGov contains information on parties, elections and cabinets for 37 countries, including all EU and most OECD democracies [10]. The CHES use expert surveys to “estimate party positioning on European integration, ideology and policy issues for national parties in a variety of European countries.” [9] Thus, these databases can be used to link differences in online elite political behavior and interaction to variables like party family, political position or offline discourse. In the supplementary information of this article is the Database codebook which gives an in-depth explanation about the variables included, in which tables they can be found, and the relationships between the tables in the database. These relationships can also be seen in Fig 1. The latest data can be downloaded from the TPD website: twitterpoliticians.org.

## 4 Analyses to demonstrate the Twitter parliamentarian database as a research tool

To illustrate the TPD’s capacity for comparative research, we use guiding questions to focus our demonstrations, limiting the time frame to the period from 1 January 2018 to 31 December 2018 for congruence.

### 4.1 Comparing countries: How do politicians use Twitter?

We begin by looking at the similarities and differences between countries in terms of Twitter use: *What are the differences between countries in parliamentarian Twitter interactions?* While Twitter offers the same functionalities to parliamentarians everywhere, how those functionalities are used varies significantly between countries. These differences may point to differences in political cooperation across countries. Firstly, the percentage of parliamentarians that actively use Twitter differs. Some countries may have an extremely high active Twitter base (99%, United States) where most parliamentarians tweet almost daily, whereas in other countries, the parliamentarians may have Twitter accounts, but they rarely tweet. However, on average 80% of parliamentarians per country are active on Twitter. The frequency of Twitter use varies across countries. On average, parliamentarians tweet 2.8 times per day, although there is some deviation; parliamentarians in Iceland tweet less than once per day, whereas parliamentarians in Turkey tweet 6 times per day (*min* = 0.7;*max* = 6.6;*σ* = 1.4).

**Fig 2:**
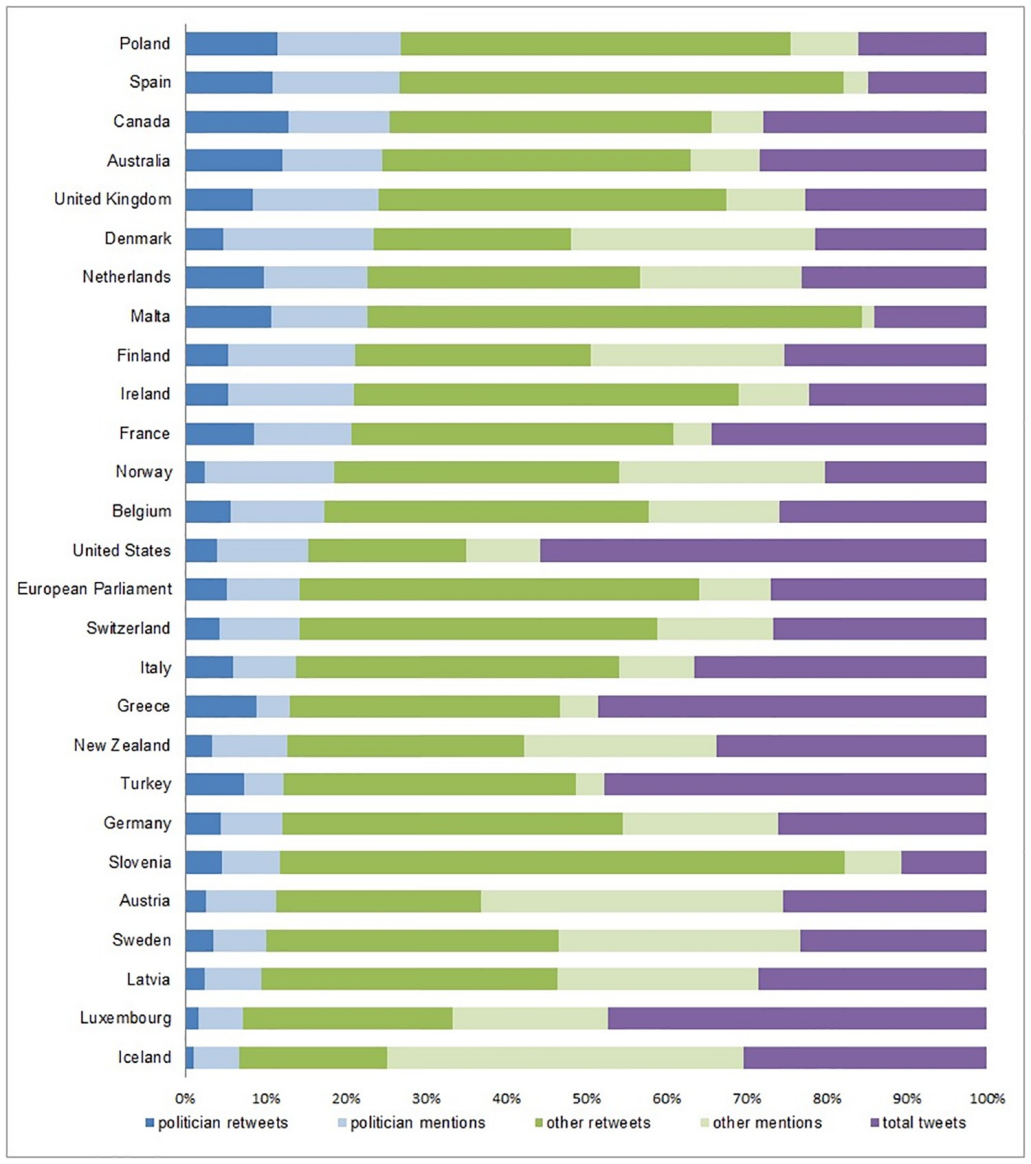
Ratio of retweets & mentions. Retweets and mentions per country as a percentage of total tweets for all countries in the database.

Retweeting and mentioning exclusively between national parliamentarians make up an average of only 21% of total politician Twitter activity (min = 7%, max = 36%), with Poland having the highest proportion of Twitter activity between their parliamentarians, and Iceland with the lowest. Further, as we see in Fig 2, while mentions to other parliamentarians tend to be used much more than retweeting, there are stark differences between countries: Icelandic parliamentarians very rarely retweet, while Greek, Turkish and Canadian parliamentarians have more retweets than mentions. Conversely, we see the opposite pattern when looking at retweets and mentions to non-parliamentarians, as parliamentarians retweet more than mention when communicating with those outside of parliament.

The data on retweets and mentions can however be employed in much more powerful ways, providing new data to questions that have long been central to political science. For instance, a large literature in political science focuses on international comparison of politician cooperation, and the relation to aspects of the countries’ democratic systems. This literature follows not least from Lijphart’s [51] suggestion that consensus democracies, usually employing proportional representation systems, leads to “kinder, gentler” political cultures than majoritarian systems (see also e.g. [63–65]). The authors postulate that the structures of power distribution represented by the democratic system of a country (e.g. majoritarian or consensus) may encourage attitude polarization (and in turn elite conflict), or instead, foster cooperation between politically dissimilar parties [51, 52, 66, 67]. With this reasoning, it is thought that proportional systems lead to increased cooperation [51]. Conversely, other scholars argue that due to lower barriers of entry for smaller, single-issue parties, political fragmentation is instead increased in proportional systems [66–69].

Academic work on the coalitions and divisions within parliamentarian communication networks has been limited by lack of suitable empirical data. The TPD can thus provide a useful data source for studying the coalitions and divisions among parliamentarians. By viewing retweets as endorsements (c.f. [70, 71]), the patterns of retweeting can be revealing of the political alliances within a country. Retweets can be treated as edges in a network, wherein the structure can reveal coalitions and divisions amongst parliamentarians. Such networks can be analysed in different ways, capturing different aspects of the structure of endorsements within a country. For this demonstration, we focus on the networks with 30 or more parliamentarians, and filter the networks by their giant component. We here aim only for a tentative exploration, leaving an in-depth and rigorous analysis for future research.

A simple but powerful way of operationalizing the level of cohesion within a country is to compare the fraction of retweets made to members of external parties. Countries whose parliamentarians frequently use retweets to endorse members of other parties can be assumed to have more amicable between-party relations compared to countries whose parties mostly retweet internally. S2 Table documents the electoral system, and the average fraction of external retweets per country. It shows that there are clear differences between countries in terms of the fraction of external retweets. Although these patterns need further exploration, we see that majoritarian (M) systems tend to have a lower fraction of external retweets compared to countries with mixed and proportional representation (PR) systems. PR systems on the other hand, show wide variation in the average fraction of external retweets. Countries with a high fraction of external RTs all employ PR systems.

However, this quantitative approach has certain limitations, as it fails to capture, for instance, divisions involving multiple parties in coalitions or situations in which a fraction of a party is strongly divided from other parties. As there are many forms that divisions can take in these networks, we turn to a more qualitative approach to network analysis: Visual Network Analysis [72]. This highly flexible approach allows us to categorize the endorsement networks according to their structure, identifying various forms of divisions and alliances within a country.

**Fig 3:**
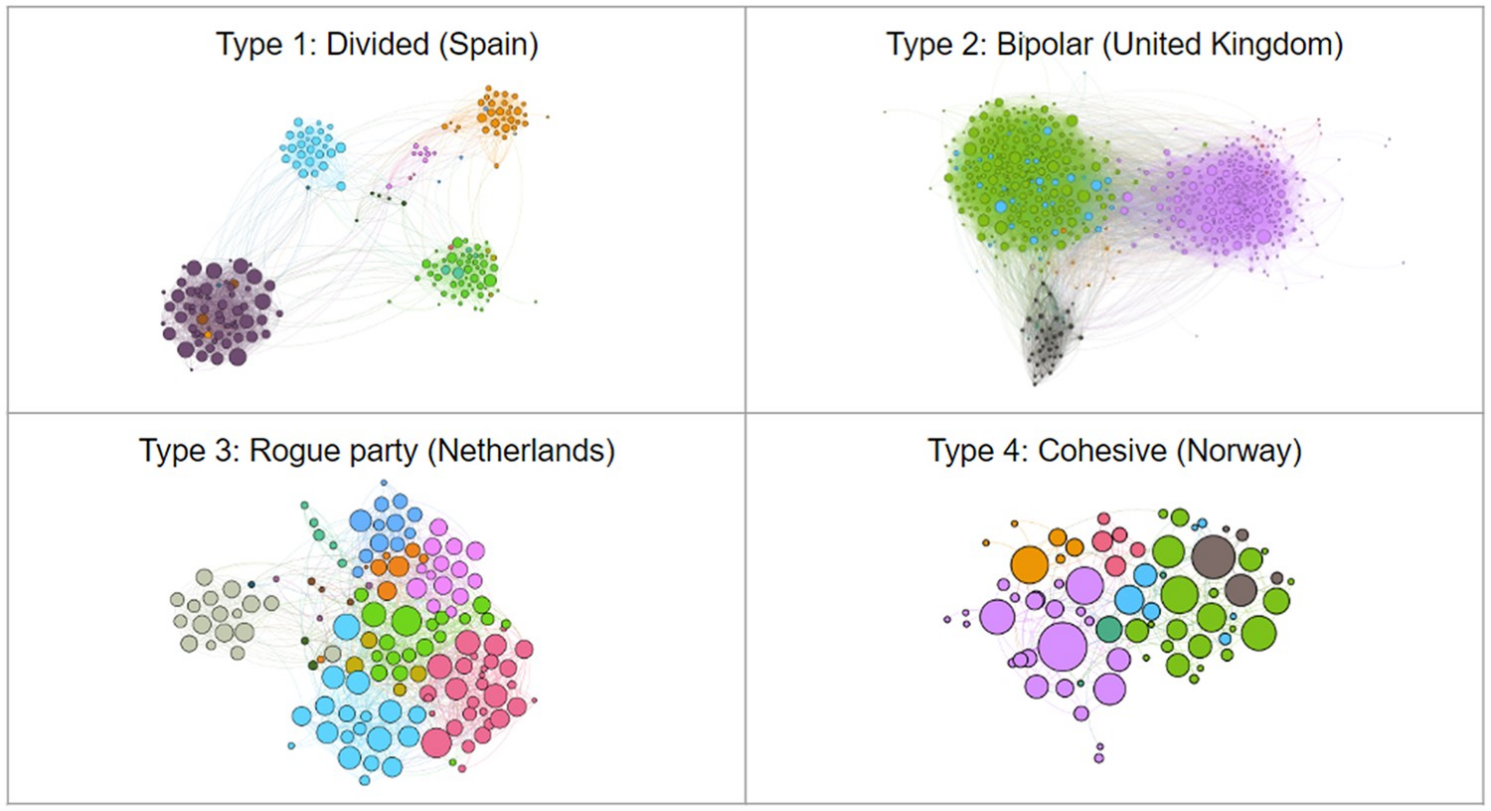
Parliamentarian retweet network archetypes. Shows the 4 distinct types of retweet networks. Each node represents an individual parliamentarian. The networks are plotted with the ForceAtlas2 visualization algorithm which plots highly connected nodes close to one another, and less connected nodes further away. The nodes are colored by party.

To look beyond descriptive metrics for examining the differences in parliamentarian twitter interactions, for this preliminary demonstration we focus on the networks with 30 or more nodes and filter the networks by their giant component. We plot the networks using the ForceAtlas2 visualization algorithm, which uses a number of properties to structure the networks in such a way that highly connected nodes tend to be closer to each other, and less connected nodes further away from each other [73]. In Fig 3, the nodes are colored according to their party.

Using this qualitative method identify four distinct types of political network structures (see Fig 3). Type 1 networks show a highly divided structure: there are clear divisions between clusters and very few cross-cutting ties. Type 2 networks show two large clusters that have dense connections amongst themselves and fewer external ties. Type 3 networks show a large, densely connected structure with one or two outlying parties, which are weakly connected to the others. A clear example of this is the Netherlands, where the outsider is radical-right party PVV and Germany, where the outsider is the radical-right AfD, which entered parliament in the 2017 election. Lastly, Type 4 structures exhibit one large cluster of dense connections. All the individual retweet networks and their classifications can be found in S1 Fig.

We can furthermore use additional measures to compare the categories of networks identified using VNA, to demonstrate that the networks are not only visually distinct. S2 Table also reports the modularity, number of clusters, and average clustering coefficient per country to provide measures of clustering within the networks. To confirm the stability of the modularity measure, we used the leidenalg python library to measure each network 1,000 times. We report the average modularity from these runs, as well as the most frequent number of clusters per run. Cluster assignment was based on the most frequent cluster assigned per node across the 1,000 runs. The results indicate that type 1 networks tend to have higher modularity, as well as a greater number of clusters than other networks. Type 3 networks tend to have fewer clusters than the other network types. Type 1 and 2 networks tend to have higher clustering coefficients whereas type 4 networks have lower clustering coefficients. This would indicate that type 1 networks tend be more divided than types 3 and 4. The fraction of external retweets also shows that parties in type 1 and 2 networks retweet other parties much less than those in types 3 and 4. Additionally we see that most type 3 networks tend have negative kurtosis of their degree distributions, which implies that members in the network retweet each other to a similar degree, rather than rallying around a few leaders. Finally, we see that type 1 networks tend to have lower normalized eigencentrality scores, implying that nodes are less well-connected within the network.

We also look at the relationship between cluster and party membership through *χ*^2^ and Cramer’s V measures, wherein Cramer’s V show the strength of that relationship, thereby indicating how ‘neatly’ the networks cluster based on party: fragemented networks are expected to have a stronger relationship between party-cluster membership, whereas a weak relationship would indicate more overall cohesion in the network. For brevity, we only report the Cramer’s V value in S2 Table, as all *χ*^2^ results indicated a significant relationship between party and cluster membership (*p* < 0.00). We see that type 1 networks have much stronger relationships between party and cluster membership than type 4 networks. Therefore, it is clear that type 4 networks generally retweet across party lines.

All in all, visual analysis and basic network measures can be combined to interpret the types of network structures that emerge from the data. The measures provided are by no means exhaustive and may differ depending on the research question. Using the information from the ESDD [8], we can explore differences in parliamentarian endorsement not only between countries, but between democratic systems. We see that Type 1 networks show a divided network with little endorsement between parties. Type 2 networks appear bipolar, with two large contending groups, and are the most common category for Majoritarian systems. Lastly, types 3 and 4 are more consensual, due to many cross-cutting endorsements beyond party lines, and are largely comprised of PR systems. These results are tentative and not exhaustive. However, they do illustrate that the database offers opportunities to compare coalitions and divisions between different countries and political systems. While our purpose here is to illustrate the potential of the dataset for studying cross-national differences through exploratory analysis, it also includes data that allows for more formal and quantitative measurement, and points to several fruitful directions for future research.

### 4.2 Comparing language between parties: Examining Hashtags

Textual data from Twitter provides an avenue into seeing how parliamentarians navigate different issues and engage in discursive conflict. Despite this potential, there has been limited research on content of parliamentarians’ tweets, and none that has focused on international comparison [56]. The TPD offers the opportunity to contribute to comparative work on discursive conflicts [57, 58] that so far had to rely on newspaper data that typically do not provide a comprehensive coverage of political claims [59] and is time-consuming to collect and process [74]. Since the TPD furthermore links to the Manifesto Project, Chapel Hill, and ParlGov databases, it has the information necessary for connecting such textual analysis to a large body of work that compares different political parties and party families [7, 75–77]. In this section, we explore the possibilities for using the TPD to take a comparative approach to the study of political discourse, focusing on the differences in the use of hashtags between political parties.

The use of labels in political discourse reveal the different ways that opposing political parties discuss the same issues. This is apparent through brief exposure to political debate: while one party may speak of “tax reform,” the other focuses on “tax relief.” Using different labels for the same issue indicates how central labelling is to politics [78]. The way an issue is labelled influences how we view that issue, what issues we see in the first place and enable us to make sense of what we are reading. Using certain labels over others may lead to exacerbating political divides: when every group identifies and labels its own issues in its own way, it makes conversations across partisan lines more difficult [79].

Analyzing labels used on Twitter is made easier by Twitter providing affordances for explicitly labeling tweets, in the form of hashtags. While hashtags serve many purposes, broadly speaking they can be used to index conversations [80], convey a particular point of view [48], or for issue positioning and labelling [25, 26, 48, 81]. Importantly, hashtags aid in building public perceptions of an issue by ensuring maximum visibility, and allow anyone to jump into the conversation [82]. In order to demonstrate the database as a tool for comparing issue labelling between parties, we therefore we look at hashtags, using the guiding question: *how do political parties differ in their use of hashtags and what is their common ground*?

**Fig 4:**
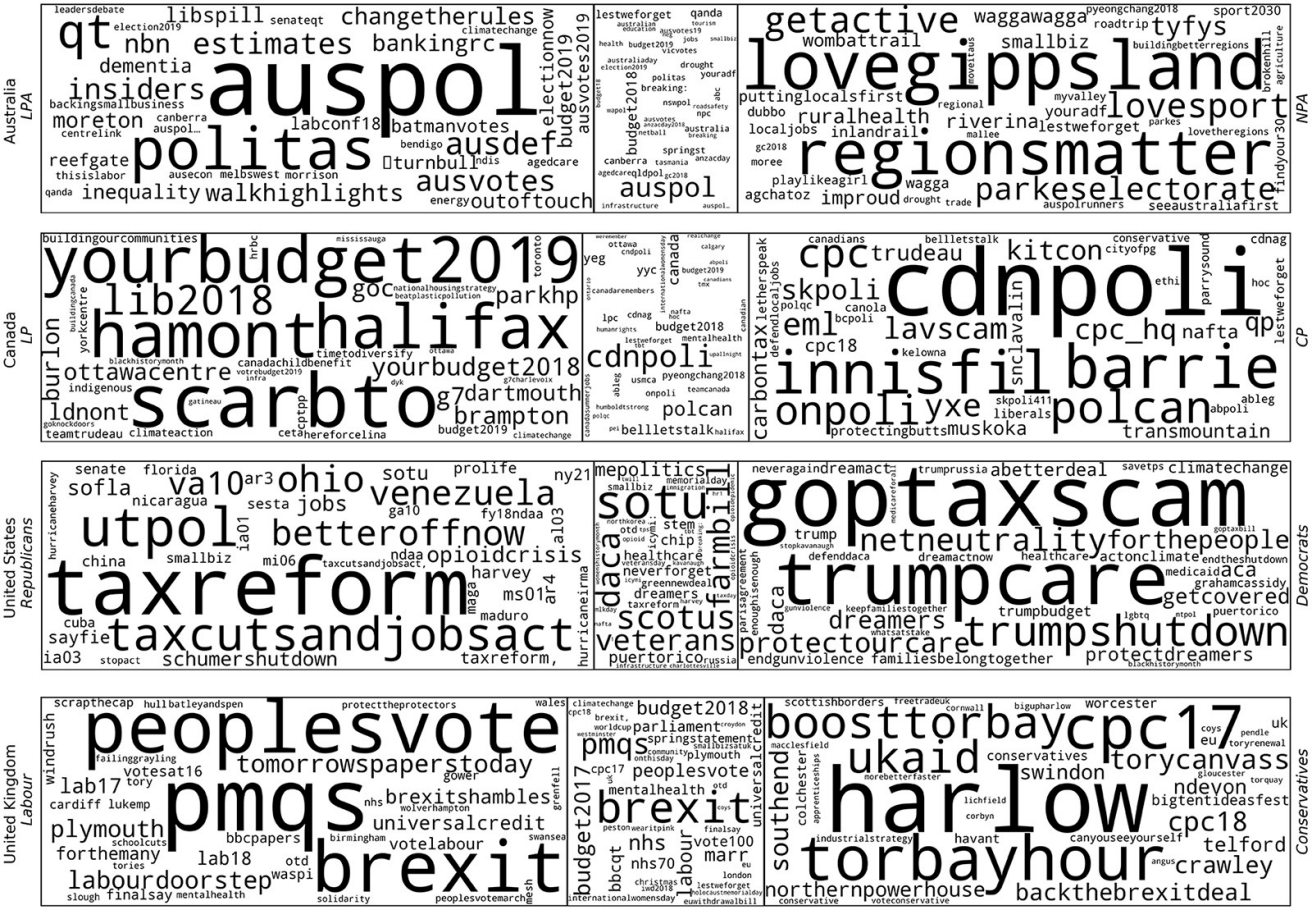
Hashtag use amongst the 2 largest parties. This figure shows partisan and common hashtags in Australia, the United States, Canada, and the United Kingdom. The middle panel shows the common ground while the side panels show the distinctive hashtags for two largest parties. The size of the middle panel is proportional to the size of the common ground relative to the parties. In the word clouds, the size of words is proportional to the frequency of use as a fraction of total hashtag use.

To take a first step toward a comparison of how different parties label issues, we develop a simple computational method that captures which hashtags are partisan and which are shared. To study this, we look at the two largest parties in all countries, in terms of the number of active users on Twitter (note that these are not necessarily the parties that have the most seats in parliament). We count each hashtag used by these parliamentarians and then normalize according to the total number of hashtags used by the party. If a hashtag is used by both parties, the overlapping part of the use is seen as the intersection between the parties. As in a Venn diagram, the intersection of the sets are the common hashtags, and the non-intersecting parts are hashtags characteristic of the party. The relative size of the intersection thereby gives a measure of the similarity in hashtag use between the two parties, which corresponds to the Jaccard similarity measure [83]. Since dynamics of discursive conflicts are likely different in multiparty systems, we here focus on the majoritarian systems categorized by the ESDD: the UK, USA, Canada and Australia. The results are shown in Fig 4. While the set-based method used here is intuitive and therefore useful for exploratory analysis, a common statistical way of comparing corpora for the most overrepresented words is Log-Likelihood [84]. This analysis can be found in S3 Table, and largely matches the results in Fig 4.

A first striking takeaway from this Fig 4 is that the common ground hashtags represent only a small fraction, where the intersection is 15.7% (in the United Kingdom) or less. Most of the hashtags that are equally represented in each party (proportional to the number of total hashtags used by that party), are usually smaller, less commonly used tags. This indicates that hashtags are largely partisan. Hence, to understand what these fractions mean, we need to look closer at the way hashtags are employed by politicians.

A first thing to note is that the most common intersecting hashtags for Canada and Australia are “cdnpoli” and “auspol” respectively, although they are not used with the same relative frequency by each party. These are general country-level hashtags for marking the nation which the discussion concerns, and are broadly employed for political debate also outside of professional politicians (these hashtags have been broadly studied, e.g. [24, 85, 86]). The UK and the US do not seem to have any corresponding hashtags, which may be due to the US and UK being large enough to be the norm on the platforms, while Canadian parliamentarians need to demarcate that they are speaking of Canadian politics.

We focus here on the US for a deeper look into the ways parties make use of hashtags, and the relation to how political issues are labelled. Looking at Fig 4, we see that politicians from both parties address many of the same issues, while using very different labels. The 2018 tax reform is a major talking-point on both sides, but while Republicans refer to it using “taxreform” or “taxcutsandjobsact,” Democrats instead use “goptaxscam”. Interestingly, there is no common ground hashtag to denote the bill. A similar state of affairs can be identified in relation to the 2018 government shutdown, which both parties attempt to attribute to the other party: Democrats refer to it as the “trumpshutdown,” while Republican use “schumershutdown”. An interesting point of note is that Democrats use the hashtag “trump,” while this is not among the major hashtags for Republicans. Republicans focus on “Venezuela,” as an example of the putative dangers of left-wing politics, while Democrats speak of “climatechange,” an issue that is not featured among top republican talking-points.

The most important intersecting hashtags found in Fig 4 include tags which describe arenas of contention rather than specific topics of disagreement, for instance, “scotus,” referring to the Supreme Court, in which highly a contested process of electing new judges played out during 2018. Another example of this is “farmbill”, referring to the primary agricultural and food policy bill of the US government, which is renewed every 5 years and deals with affairs under the purview of US Department of Agriculture. While there may be disagreements about its contents, the common use hashtag suggests that representatives from both parties at least agree they are discussing the same issue.

Other important intersecting hashtags point to common ground values, to which both parties are happy to profess their support. These include “veterans,” “neverforget,” and “stem”. Similar common ground is found in national emergencies, such as the hurricane striking Puerto Rico. However, even here the language differs somewhat between the parties: Republicans are more likely to refer to the event as “hurricaneirma”, while Democrats speak of its impact on “PuertoRico”. DACA (Deferred Action for Childhood Arrivals)—an Obama-era executive action, now turned Trump-vetoed bill with broad bipartisan support among voters—is an important hashtag in the intersection, but is driven in particular by Democratic politicians, who also employ the related hashtags “dreamact”, “dreamers”, “protectdreamers”. There is, perhaps somewhat tellingly, broad bipartisan use of the hashtag “mepolitics,” denoting a criticism of the country’s polarized political discourse.

As we have demonstrated, we see that politicians use hashtags to express partisan claims. While there is some common ground (for instance when it comes to the importance of caring for veterans), politicians generally use hashtags in outspokenly partisan ways: they use specific hashtags to push different kinds of issues on the agenda or to express a partisan take on the same issue. This case has served to illustrate the range of possible research opened by the TPD in examining language and discourse of politicians on Twitter.

### 4.3 Transnational communication: What is the structure of the transnational mention network?

The TPD not only allows for cross-national comparative research but also for research on international and transnational politics, by enabling analysis of how parliamentarians of different countries communicate with one another [87–89]. Through facilitating communication and mobilization of opinion across borders [90], social media platforms open up for the possibility of a cross-national dialogue. The TPD allows us to examine systematically and comprehensively whether parliamentarians are employing the affordances of the platform to engage in transnational debate. To demonstrate, we look at all mentions between politicians in the European Free Trade Association (EFTA). When a parliamentarian uses the Twitter mention functionality, it refers to another Twitter user and notifies the user that they have been tweeted about. Mentions in this case are tweets in which another parliamentarian’s Twitter account is signalled with the ‘@’ symbol. This does not include direct retweets, but it does include retweets where additional text has been added. Mentions were chosen over retweets as they are more indicative of a dialogue or debate (rather than an endorsement), as well as to provide illustration of analysis of Twitter’s affordances beyond retweets in the TPD. Luxembourg and Slovenia were excluded from the analysis due to their low number of international mentions. Moreover, since we are interested in communication between national parliamentarians, we do not include the European Parliament in this analysis (see [91–93] for studies on the European Parliament). As illustrative questions, we ask; *what is the structure of interaction between parliamentarians in the transnational debate*?

**Fig 5:**
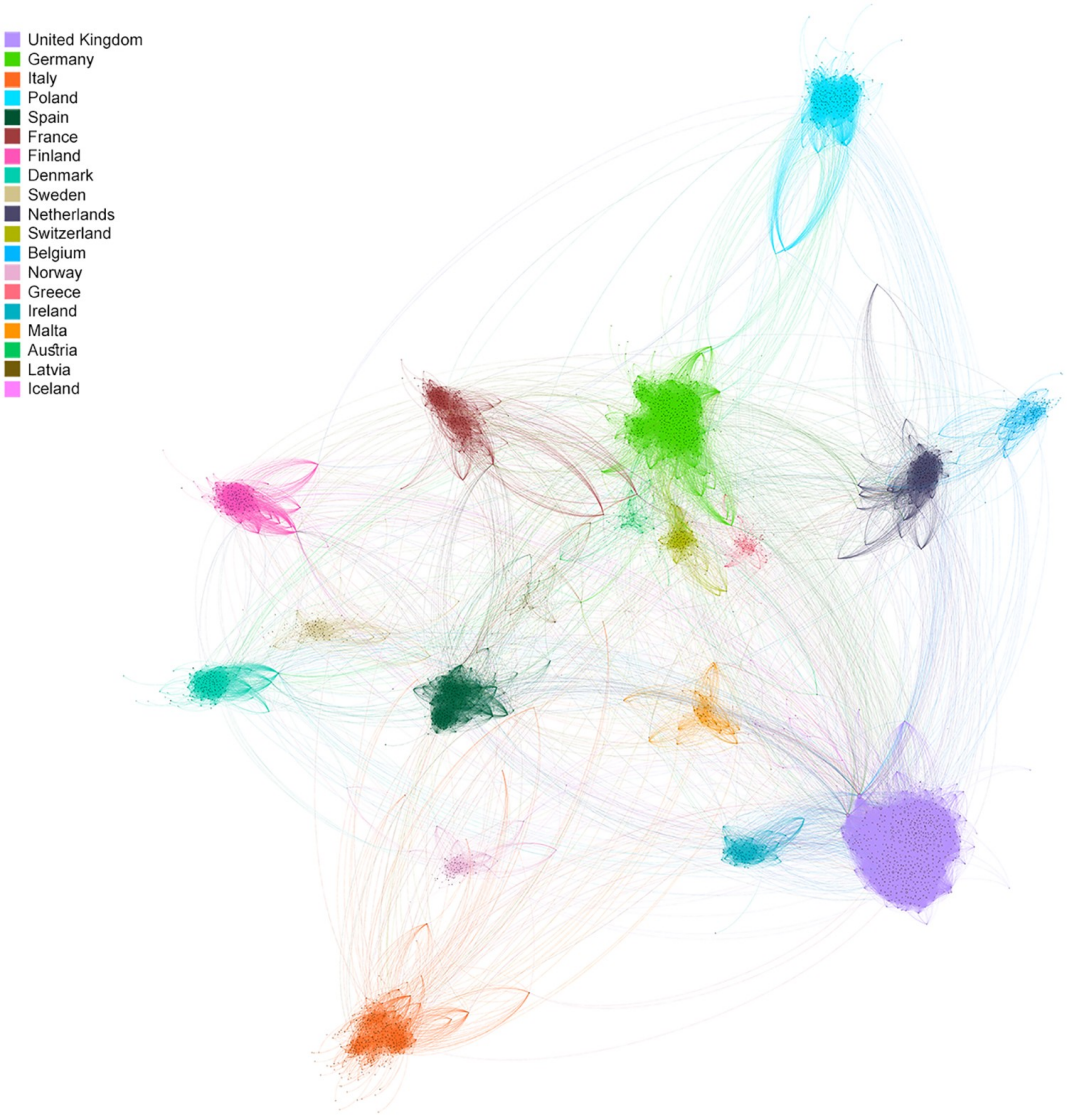
International mention network. This network shows all mentions between parliamentarians in the database. The network is made in Gephi, using the ForceAtlas2 algorithm. Node colors are set according to country.

We begin by examining the network of mentions between individual parliamentarians, filtered by its giant component (see Fig 5). In most countries (with the exception of Ireland and Spain), less than 55% of parliamentarians have mentioned a parliamentarian from another EFTA country. S4 Table contains the proportion of parliamentarians that made external retweets per country. The extent to which the international network is split along national lines is striking: the parliamentarians are organized in clusters that almost perfectly cut along national lines, with only a small number of parliamentarians being located outside their respective national cluster. Moreover, when there are connections between national clusters, they tend to occur between neighboring countries, with nations like Ireland and UK situated next to one other in the network.

To arrive to a more precise measure for quantifying the level of external communication between countries, we look at the fraction of external tweets, network diameter, average path length and modularity. The modularity indicates the strength of the division of the network. The network diameter measures the longest distance between any two nodes in the network, whereas average path length counts the average graph distance between all pairs of nodes. These measures help to understand the structural connectivity amongst individual national parliamentarians on a European level. Aggregating on country level, we examine centrality with PageRank. The network diameter is 18 and the average path length is 5.8, indicating that there is quite some distance between distant nodes in this network. The network has a modularity of 0.678, suggesting that the network is relatively divisible into separate clusters. All these numbers suggest that parliamentarians communicate mostly with their compatriots, resulting in a very sparse and highly divided international communication network.

**Fig 6:**
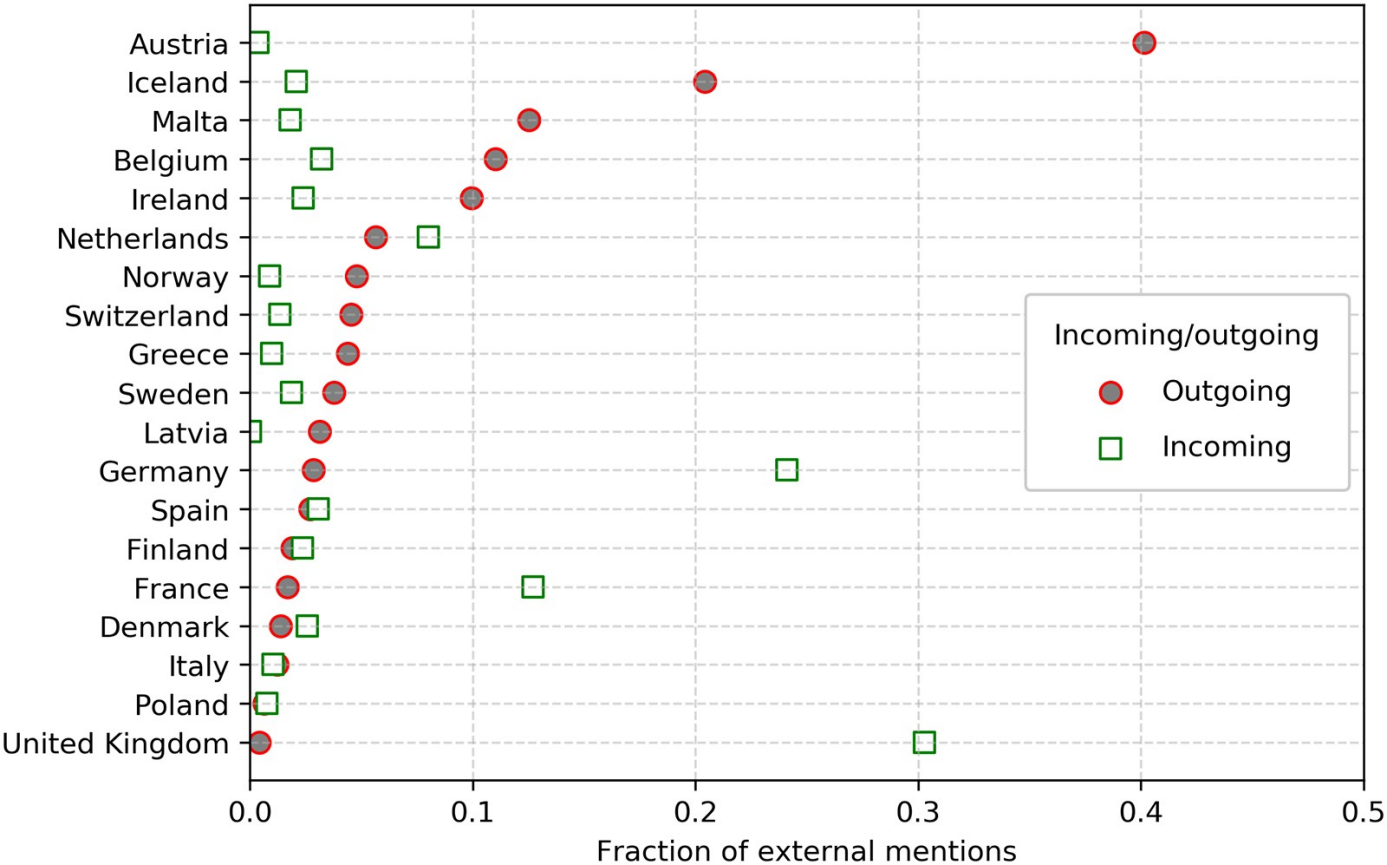
Incoming and outgoing mentions. Shows the normalized fraction of outgoing and incoming mentions. The fraction of incoming mentions shows which countries are more central to the debate whereas the outgoing mentions shows how much mention activity per country is directed to international debate.

Although international communication accounts for a low proportion of the total communication (1.9% of all mentions are directed to a parliamentarian in a different country), in absolute terms it is still substantial cross-national traffic (16,955 total mentions). Looking closer at these mentions gives insight in patterns of communication across EFTA countries. We examine the fraction of external mentions per country, to determine the proportion of mentions that are dedicated to international debate [94]. When looking at fractions of external mentions, we see in Fig 6 that some countries participate minimally in international debate, through a low fraction of incoming and outgoing international mentions. Moreover, we see that the majority of all international mentions are directed towards a small number of countries, namely the United Kingdom and Germany, and to a lesser extent, France. Additionally, we see that there is a large imbalance in outgoing and incoming mentions for some smaller parliaments such as Austria and Iceland. Small countries appear relatively outward facing, as there are simply fewer parliamentarians to mention within these small countries, and more external parliamentarians, resulting in a higher fraction of external mentions. Moreover, it is noted that smaller countries tend to be more globalized than larger countries [95].

**Fig 7:**
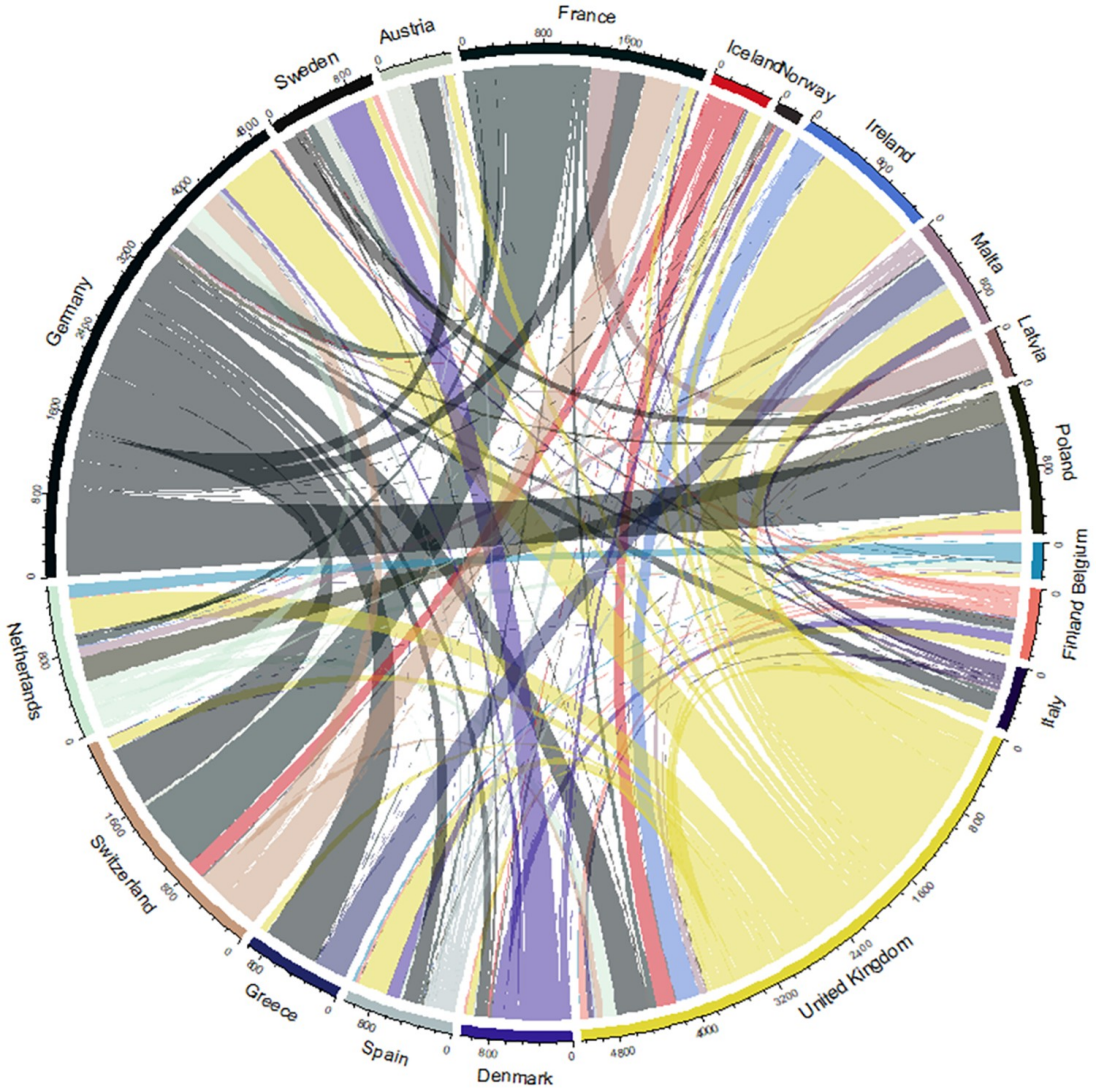
Incoming and outgoing mentions. Shows the fraction of outgoing and incoming mentions per country. It includes only international mentions, thereby showing the importance of each country in the international debate.

**Fig 8:**
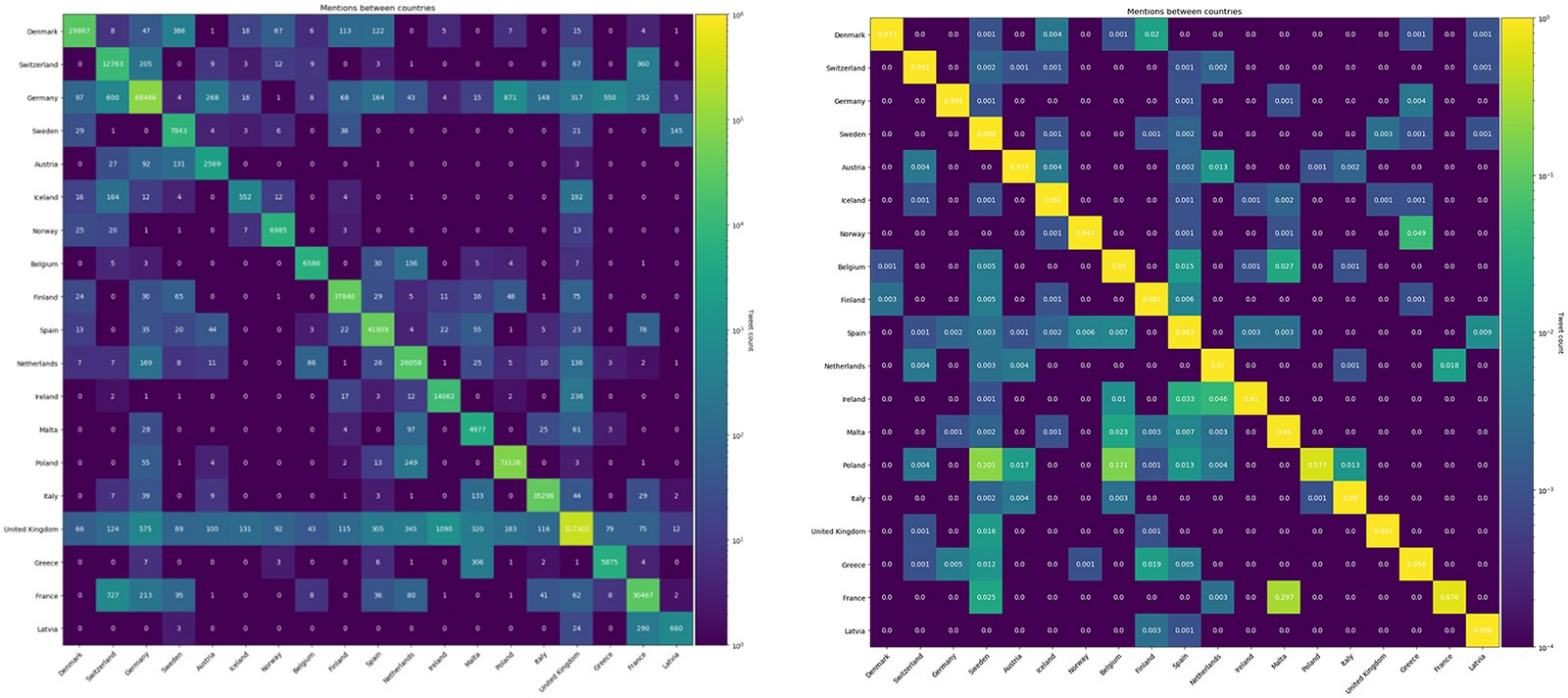
Absolute and normalized adjacency matrices. Shows absolute (left) and normalized (right) adjacency matrix for the mention network. (Note that the color scheme is logarithmic).

**Fig 9:**
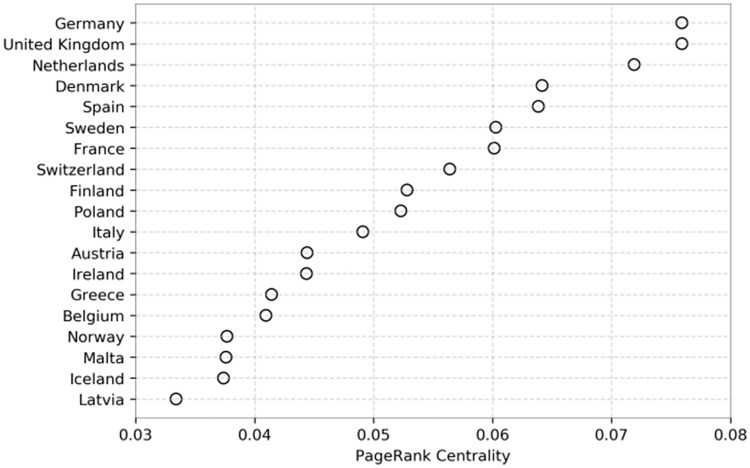
PageRank centrality per country. To investigate which countries are most central in the international debate in Europe, this figure shows the PageRank centrality of the aggregated network.

To further examine the extent of the transnational debate, Figs 7 and 8 show that the international debate is uneven, being dominated by Germany and United Kingdom. To check if countries contribute equally to the international debate, we used an independent samples Kruskall-Wallis test. We compared the relationship between normalised cross-border tweets and country. The results indicate that some countries have significantly more external ties than others (p = 0.000). This implies that some countries participate more than others in the transnational debate. For a more precise estimate of the relative importance of different countries, we calculate PageRank centrality on the adjacency matrix of the aggregated connections between countries. The results are shown in Fig 9. The figure shows that while France has a large and active parliament, they are relatively peripheral in the international debate compared to Sweden, Denmark and the Netherlands—all whom are significantly smaller in parliamentary size but higher in centrality. The sheer volume of mentions emitted by UK parliamentarians—gives it a central place in the transnational debate as shown by its PageRank. Germany, by contrast, has significantly fewer external mentions but are are just as central in terms of PageRank and relatively more connected, which is also shown by their fraction of incoming external mentions. Thus, Germany also occupies a central position in European debates.

In summary, the results of our demonstration suggest that debates among national parliamentarians remain, by and large, contained within national boundaries. The TPD not only makes it possible to study the degree of transnational communication, but also to examine the position of countries, parties, or individual parliamentarians within that debate and the role of different issues. While our exploratory analysis has shown which countries are more central to transnational debates within Europe, the TPD makes it possible to study transnational politics on Twitter at a grater scale and in more detail than data sources have previously allowed for.

## 5 Conclusion

This paper presents a database that responds to the methodological issues regarding delineation, sampling and validation methods commonly used in Twitter research, using a variety of data sources along side manually validated Twitter information. The opportunities of this database for comparative and transnational research were illustrated through three tentative studies, looking at 1) national differences between parliamentarians’ Twitter use, 2) differences between political parties’ hashtag use and 3) the structure of transnational debates.

Through our exploratory analyses, we discover similarities in the ways which politicians use Twitter across countries. Overall when communicating with other politicians, they prefer to use mentions rather than retweets. There are also many differences: some countries have a very active, thriving Twitter culture in which all functionalities are used, others refrain from mentioning and retweeting, and yet others may not use the platform much at all. Apart from documenting differences in how Twitter is used, the database can also provide an empirical foundation for research into long-standing questions in political science. To illustrate the database as a tool for studying these questions, we used the structure of retweet networks as a proxy for coalitions and divisions in parliamentarian Twitter networks. Using Visual Network Analysis, we could distinguish four different kinds of retweet networks: bipolar, fringe party and cohesive. These structures show whether or not there is a lot of endorsement across party lines, or if the political culture seems more strictly partisan. Indeed, when looking at clustering measures, we see that networks that appear more divided have a higher clustering coefficient. We find that majoritarian systems have less external retweets and higher clustering coefficients, and most commonly resulted in bipolar structures. On the other hand, proportional systems are the only systems that resulted in cohesive networks. This suggests that there is a correspondence between the nature of political system and patterns of political communication but further investigation is necessary to arrive at robust conclusions.

Comparisons between parties are made through using hashtags as a conduit for issue labelling. We compare countries with majoritarian systems, as they have two large opposing parties that comprise the bulk of the politicians’ Twitter activity. We find that there are very few hashtags that are shared between the opposing parties, and, in line with existing literature [26, 96, 97], hashtags are used by politicians for issue positioning. The TPD is able to be used when looking at the content of parliamentarian tweets to determine which party used which hashtag, which can help provide a clear identification of partisan and shared tags within a country. This identification can then highlight issues of importance between different parties. Thus, hashtags are an interesting future avenue to study how parties label and discuss issues.

The TPD does not only allow comparisons of countries and parties but also the study of transnational communication. By way of illustration, we studied cross-European parliamentarian communication. We found that cross-national mentions constitute only a tiny portion of the total politician Twitter use, which confirmed by the fraction of external mentions per country. Therefore there is very little international interaction amongst national parliamentarians. Additionally, through PageRank measures we can see that Germany and the United Kingdom take central positions in these debates. Hence, we have demonstrated that the TPD is able to explore the degree in which cross-European communication exists for parliamentarians on Twitter, along with the position of the countries, parties or individual parliamentarians across a number of different issues with more detail than previous studies.

This paper has therefore demonstrated that the TPD is a powerful database for carrying out research on parliamentarians’ use of Twitter, in particular for cross-country comparative and transnational research, which has thus far struggled with data availability. All in all, our database addresses some of the current methodological issues with Twitter research and provides a starting point for studying communication, contention and cooperation not only within countries, but also comparatively across borders. As far as we are aware, the TPD is the most comprehensive database of parliamentarians on Twitter that exists, and it is able to provide a framework for more standardized comparative methodology in politician Twitter research.

## Supporting information

S1 File The database codebook. (PDF)

S1 Fig Retweet networks per country, 2018. Shows the individual country retweet networks for 2018. The nodes are colored by party and the network is visualized with the ForceAtlas2 algorithm, with each node sized by in-degree (1-10) and scaling set to 1.
(TIF)

S1 Table This table shows the number of MPs on Twitter per country in the database. The legislative period id is an arbitrary id number that distinguishes each legisla-tive period.
(PDF)

S2 Table This table shows clustering and degree measures, as well as fractions of external mentions and the strength of relationship between party and cluster membership, applied to the individual country networks. (PDF)

S3 Table This table shows the highest Log-Likelihood values (shown in brackets) of hashtags used by the two largest parties in 2018. High values indicate that a term occurs more frequently than chance amongst one party.
(PDF)

S4 Table This table shows the number and percentage of MPs who mentioned another politician in the EFTA in 2018. (PDF)
