# Supplementary material for: The Twitter parliamentarian database: Analyzing Twitter politics across 26 countries
Source: PLoS One. 2020 Sep 16;15(9):e0237073. doi: 10.1371/journal.pone.0237073 (PMC7494116; doi:10.1371/journal.pone.0237073)
Supplement: S1 File — (PDF) [file pone.0237073.s001.pdf]

Database Codebook

Contents

- Data sources
- Tables
  - Summary of all tables
  - Individual tables
    - Members
    - legislative_periods
    - countries
    - parties
    - political_groups
    - Parties_political_groups
    - Parlgov_parties
    - Mp_data
    - ches2014
    - ches2017
    - tcat_tweets
    - tcat_mentions
    - tcat_hashtags
- Index
  - Government websites
- Example Queries

Data sources

- Government websites - see index for all websites scraped
- Twitter
- Electoral system design database (https://www.idea.int/data-tools)
- Manifesto Project Database (https://manifesto-project.wzb.eu/)
- Parlgov database (http://www.parlgov.org/)
- Chapel Hill Expert Survey (https://www.chesdata.eu/)
- Manual addition of electoral periods and legislative period data

Tables

Summary of all tables

**Table name**	**Description**
members	All of the member names, taken from official government websites
legislative_periods	The legislative period that the country is in and its electoral details
countries	The countries of which parliamentarians on twitter which are included in the study
parties	The political parties
political_groups	Political groups - only applicable to some cases. Some nations emphasise political groups (coalitions) over party membership
parties_political_groups	This table is the mediating table for the many-to-many relationship between parties and political groups. Parties can change political groups between legislative periods.
parlgov_parties	The database from parlgov.org
mp_data	The database from the manifesto project
ches2014	Data from the Chapel Hill Expert Survey 2014
ches2017	Data from the Chapel Hill Expert Survey 2017
tcat_tweets	Tweet_ids and uids
tcat_mentions	All the tweet_ids from retweets and mentions

tcat_hashtags	All the hashtags & their tweet_ids

Individual tables

Members

The members table can have the same person with two different member ids, but one twitter uid.

- This is because some members change parties per legislative period

column name	format	description
member_id	int	Primary key identifying each member per legislative period
name	varchar	The name of the politician
party_id	int	Foreign key linking to the parties table
pol_group_id	int	Foreign key linking to the political_groups table
party_pol_group_id	int	Foreign key linking to the parties_political_groups table
chamber	varchar	Whether or not the member is in parliament or the senate
uid	int	The twitter user id
party	varchar	The party name: for readability
name_link	varchar	A link to the individual profile of the member on the government website
function	varchar	The function of the member
region	varchar	Which region the member serves
constituency	varchar	Which constituency the member serves
scraper_url	varchar	The url from the government website that the data was scraped from.
date_of_inactivity	varchar	If a member becomes inactive before the official term ends
country	varchar	Name of the country, for usability

country_id	int	The foreign key linking to the countries table
legislative_period_id	int	The foreign key linking to the legislative_periods table
mp_party_id	int	Links members to mp_data, ches2014, ches2017 and parlgov_parties

legislative_periods		
**Column name**	**format**	**description**
legislative_period_id	int	Primary key identifying each unique legislative period per country.
country_id	int	Foreign key linking to country table
country	varchar	For usability
official_legislative_period	int	(If available) The official legislative period of the country. *Note: Some countries don't count this*
assigned_legislative_period	int	The legislative period of the country that we have since collection (i.e if it is the first or second [maybe 3rd but that'd be rare] legislative period of the country that we have in the database).
legislative_size	int	The number of people in parliament. From the electoral system design database.
legislative_voting_size	int	The number of people in parliament that vote on legislature. Usually this is the same as the legislative_size.
legislative_start_year	varchar	Starting year of the legislative period
legislative_end_year	varchar	End year of the legislative period
start_election_date	date	The election date of the legislative period
upcoming_election_date	date	The upcoming election date to start the new legislative period
legislative_start_date	date	The date that the new parliament is formed (sometimes differs vastly from the election_date)
legislative_end_date	date	The date that the current parliament is no longer seated (overlaps with the date that the new legislative

		period starts)
chamber	varchar	Whether the entry refers to the parliament or senate
electoral_system_family	varchar	From the electoral system design database.
electoral_system_for_national_legislature	varchar	From the electoral system design database.
tiers	int	The number of legislative voting tiers. From the electoral system design database.
electoral_system_for_president	varchar	From the electoral system design database.
is_current	bool	Whether the legislative period is currently active.

countries		
**Column name**	**format**	**description**
id	int	Primary key
country	varchar	Name of the country
country_abbr	varchar	2 character abbreviation for the country
lower_chamber_name	varchar	Name of the lower chamber (if applicable)
upper_chamber_name	varchar	Name of the upper chamber (if applicable)

parties		
**Column name**	**format**	**description**
party_id	int	Primary key
party	varchar	Party name
party_abbr	varchar	Abbreviation for the party (e.g. PVV)
country	varchar	For usability
country_id	int	Foreign key linking to country table

mp_party_id	int	Foreign key linking to the manifesto project table
is_nationalist	bool	(From Wiki) Indicates nationalist parties

**political_groups**		
Column name	format	description
pol_group_id	int	Primary key
political_group	varchar	Political group name
legislative_period_id	int	Foreign key to legislative_periods table
country	varchar	For usability
country_id	int	Foreign key to countries table

Parties_political_groups

The parties_political_groups table is the mediating table for the many-to-many relationship between parties and political groups.

- One party can belong to different political groups depending on the legislative period and country (e.g. lots of countries have a ‘green’ party)
- Some parties switch political groups with a new legislative period

**Column name**	**format**	**description**
party_pol_group_id	int	Primary key
party_id	int	Foreign key linking to the party table
party	varchar	For readability
country	varchar	Easier querying
country_id	int	Foreign key to countries table
political_group	varchar	For readability
Political_group_id	int	Foreign key linking to political_groups table

Parlgov_parties<br>The parlgov_parties table is the database downloaded from the parliaments and governments database		
**Column name**	**format**	**description**
cmp	int	Foreign key linking to mp_data table (links to the mp_party_id), can also be linked to mp_party_id in parties table
For other columns see: http://www.parlgov.org/#documentation		

Mp_data<br>The mp_data table is the database downloaded from the manifesto project		
**Column name**	**format**	**description**
mp_party_id	int	Foreign key linking to parties table
For other columns, see: https://manifesto-project.wzb.eu/tutorials/main-dataset		

ches2014<br>The ches2014 table is the database downloaded from the chapel hill expert survey		
**Column name**	**format**	**description**
mp_party_id	int	Foreign key linking to parties table
For other columns, see: https://www.chesdata.eu/2014-chapel-hill-expert-survey		

ches2017<br>The ches2017 table is the database downloaded from the chapel hill expert survey		
**Column name**	**format**	**description**
mp_party_id	int	Foreign key linking to parties table
For other columns, see: https://www.chesdata.eu/1999-2014-chapel-hill-expert-survey-ches-trend-file-1		

tcat_tweets		
**Column name**	**format**	**description**
id	int	Tweet id, primary key
created_at	date	Date the tweet was created
from_user_name	varchar	Screenname of the user
from_user_id	int	Twitter id of the user<br>Foreign key: links to members table
to_user_id	int	The id of the person being mentioned (in the case of @mention)
to_user_name	varchar	The screenname contained in the @mention
in_reply_to_status_id	int	Original tweet id if the tweet is a reply to another

tcat_mentions		
**Column name**	**format**	**description**
id	int	Primary key
from_user_name	varchar	The person who is doing the retweeting
to_user	varchar	Source account of the original retweet
tweet_id	int	Tweet id from twitter
created_at	date	Date the tweet was created
from_user_id	int	Twitter id of the user<br>Foreign key: links to members table
to_user_id	int	User id of the original retweet<br>Foreign key: links to members table
is_retweet	bool	1 for retweet, 0 for mention
from_leg_period	int	Legislative period of the retweeter/mentioner<br>Foreign key: links to legislative_periods table (legislative_period_id)

to_leg_period	int	Legislative period of the retweeted/mentioned<br>Foreign key: links to legislative_periods table (legislative_period_id)

**tcat_hashtags**		
**Column name**	**format**	**description**
id	int	Primary key
tweet_id	int	Id of the tweet that contains the hashtag
created_at	date	Date the tweet was created
from_user_name	varchar	The person who is tweeting
from_user_id	int	Twitter id of the user<br>Foreign key: links to members table
text	varchar	The hashtag only

Index

Government websites

Country	URL
Austria	https://www.parlament.gv.at/WWER/SUCHE/index.shtml?jsMode=&xdocumentUri=&NAME_TYP_ID=1201&NAME=&R_ZEIT=ALLE&listeId=1&LISTE=Suchen&FBEZ=FW_001
France	http://www2.assemblee-nationale.fr/deputes/liste/tableau
Germany	https://www.bundestag.de/en/members
Belgium	http://www.dekamer.be/kvvcr/showpage.cfm?section=/depute&language=nl&cfm=/site/wwwcfm/depute/cvlist54.cfm
Denmark	http://www.thedanishparliament.dk/searchResults.aspx?pageSize=100&pageNr=1#search
Finland	https://www.eduskunta.fi/EN/kansanedustajat/nykyiset_kansanedustajat/Pages/default.aspx
Greece	http://www.hellenicparliament.gr/en/Vouleftes/Stoicheia-Epikoinonias-Vouleftton/
Italy (17th)	http://www.camera.it/leg17/28
Latvia (12th)	http://titania.saeima.lv/personal/deputati/saeima12_depweb_public.nsf/deputies?OpenView&lang=EN&count=1000
Luxembourg	http://www.chd.lu/wps/portal/public/Accueil/OrganisationEtFonctionnement/Organisation/Deputes/DeputesEnFonction
Malta	http://www.parlament.mt/membersofparliament-13thlegmain
Poland	http://www.sejm.gov.pl/Sejm8.nsf/poslowie.xsp?type=A
Spain	http://www.congreso.es/portal/page/portal/Congreso/Congreso/Diputados?_piref73_1333056_73_1333049_1333049.next_page=/wc/menuAbecedarioInicio&tipoBusqueda=completo&idLegislatura=12
Slovenia	https://www.dz-rs.si/wps/portal/en/Home/ODrzavnemZboru/KdoJeKdo/PoslankeInPoslanci/PoPoslanskihSkupinah/!ut/p/z1/04_Sj9CPykssy0xPLMnMz0vMAfIjo8zivT39gy2dDB0N_C0N3Qw8Q43dTYx9QwwMvIz0w_EqMDbUj8Ij7WRMQL-HEWX6Q4nUb4ADOBpQZj9QQRRF4Ueh_70g-vF4ryA3FAIcFRUB3jOJVg!!/dz/d5/L2dBISEvZ0FBIS9nQSEh/

Iceland	https://www.althingi.is/altext/cv/en/
Norway	https://www.stortinget.no/no/Stottemeny/kontakt/Partier-og-representanter/Representantenes-e-postadresser/
Switzerland	https://www.parlament.ch/en/ratsmitglieder
Sweden	http://www.riksdagen.se/en/members-and-parties/
Ireland	http://www.oireachtas.ie/members-hist/default.asp?housetype=0&HouseNum=32&disp=mem
UK	http://www.parliament.uk/mps-lords-and-offices/mps/
Netherlands	https://www.tweedekamer.nl/kamerleden_en_commissies/alle_kamerleden
Italy (18th)	http://www.camera.it/leg18/28?lettera=A
New Zealand	https://www.parliament.nz/en/mps-and-electorates/members-of-parliament/
Turkey	https://www.tbmm.gov.tr/develop/owa/milletvekillerimiz_sd.liste
EU Parliament	http://www.europarl.europa.eu/meps/en/full-list.html
US House	http://www.house.gov/representatives/#byName
US Senate	https://www.senate.gov/senators/contact/
Australia	https://www.aph.gov.au/Senators_and_Members/Parliamentarian_Search_Results?expand=1&q=&mem=1&par=-1&gen=0&ps=100&st=1
Canada	https://www.ourcommons.ca/Parliamentarians/en/members
Latvia (13th)	https://titania.saeima.lv/personal/deputati/saeima13_depweb_public.nsf/deputies?OpenView&lang=EN&count=1000

Example Queries

Note: in order for these queries to work, you will have to create your own table of re-hydrated tweets from the tweet_ids.csv

Count number of tweets per politician

```
select
	from_user_id, count(*),
	country
from
	tcat_tweets tt
where
	tt.created_at between '2018-01-01' and '2018-12-31'
group by
	uid,
	country;
```

Getting a RT network for Australia

```
#get node data
select
	m.uid
	m.name as label,
	p.party,
	m.country,
	concat(m.country, ' ', lp.chamber, ' ', lp.legislative_period_id) country_chamber_period,
	concat(m.country, ' ', p.party) country_party,
	lp.legislative_period_id,
	m.party_pol_group_id,
	m.constituency,
	mp.corpusversion,
	mp.mp_country,
	mp.rile,
	mp.is_latest,
	mp.is_current,
	mp.oecdmember,
	mp.eumember,
	mp.mp_party_id,
	mp.partyname,
	mp.partyabbrev,
	mp.parfam,
	lp.electoral_system_family,
```

```
            lp.chamber,
            p.is_nationalist
from
            members m,
            parties p,
            legislative_periods lp,
            mp_data mp
where
            m.party_id = p.party_id
            and mp.mp_party_id = p.mp_party_id
            and m.country_id = p.country_id
            and lp.legislative_period_id = m.legislative_period_id
            and mp.is_current = TRUE
            and m.uid is not null
            and m.country = 'Australia';

#get edge data
select
            tm.from_user_id,
            tm.to_user_id,
            tm.from_country_id,
            tm.to_country_id,
            tm.from_leg_period,
            tm.to_leg_period,
            count(1) cnt
from
            tcat_mentions tm
INNER JOIN legislative_periods lp on
            lp.legislative_period_id = tm.from_leg_period
INNER JOIN legislative_periods lp2 on
            lp2.legislative_period_id = tm.to_leg_period
where
            tm.is_retweet = true
            and extract(year
from
            tm.created_at) = 2018
            and tm.from_chamber = 'Parliament'
            and tm.to_chamber = 'Parliament'
            and country = 'Australia'
group by
            from_user_id,
            to_user_id;
```
