# Supplementary figures and images for: The Twitter parliamentarian database: Analyzing Twitter politics across 26 countries
Source: PLoS One. 2020 Sep 16;15(9):e0237073. doi: 10.1371/journal.pone.0237073 (PMC7494116; doi:10.1371/journal.pone.0237073)

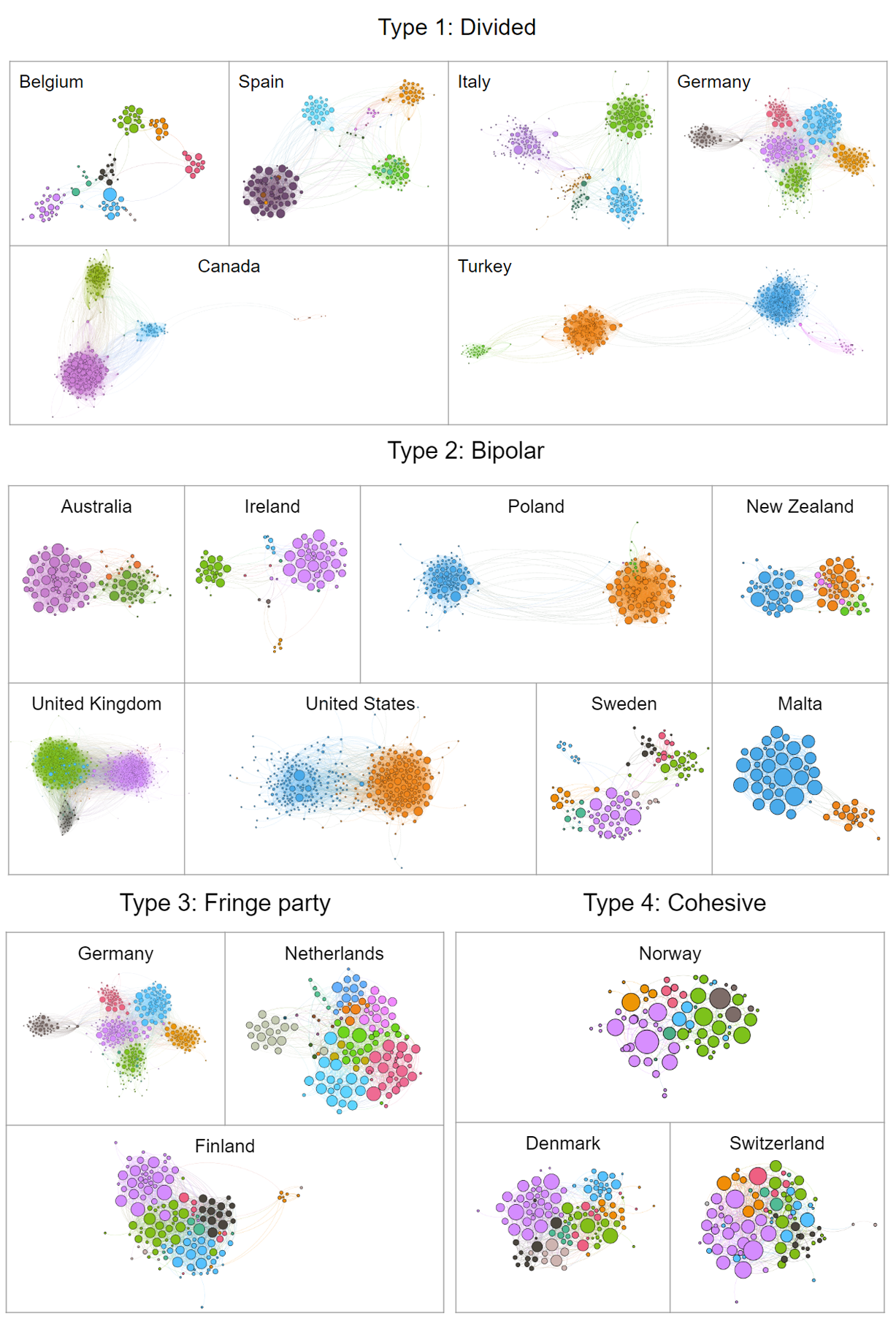

Supplement: S1 Fig — Shows the individual country retweet networks for 2018. The nodes are colored by party and the network is visualized with the ForceAtlas2 algorithm, with each node sized by in-degree (1-10) and scaling set to 1. (TIF) [file pone.0237073.s002.tif]
