# Supplementary material for: The Twitter parliamentarian database: Analyzing Twitter politics across 26 countries
Source: PLoS One. 2020 Sep 16;15(9):e0237073. doi: 10.1371/journal.pone.0237073 (PMC7494116; doi:10.1371/journal.pone.0237073)
Supplement: S1 Table — The legislative period id is an arbitrary id number that distinguishes each legisla-tive period. (PDF) [file pone.0237073.s003.pdf]

S1 Table. This table shows the number of MPs on Twitter per country in the database. The legislative period id is an arbitrary id number that distinguishes each legislative period.

Country	Legislative period id	Total MPs (N)	MPs on Twitter (N)	MPs on Twitter (%)
Australia	38	149	134	90
Austria	54	183	86	47
Austria	37	183	65	36
Belgium	35	150	130	87
Canada	34	334	327	98
Denmark	33	186	161	87
Denmark	45	185	157	85
European Parliament	32	750	635	85
European Parliament	43	747	639	86
Finland	31	202	173	86
Finland	42	199	177	89
France	30	575	515	90
Germany	28	662	355	54
Germany	29	707	511	72
Greece	27	299	102	34
Iceland	26	64	40	63
Ireland	25	158	150	95
Italy	23	637	525	82
Italy	24	630	449	71
Latvia	21	100	41	41
Latvia	22	100	57	57
Luxembourg	19	60	37	62
Luxembourg	20	45	32	71
Malta	18	67	58	87
Netherlands	17	151	147	97
New Zealand	15	119	104	87
New Zealand	16	110	100	91
Norway	14	168	114	68
Poland	13	460	334	73
Slovenia	11	90	47	52
Slovenia	12	91	54	59
Spain	10	325	219	67
Sweden	8	349	163	47
Sweden	9	357	237	66
Switzerland	6	200	123	62
Turkey	4	544	482	89
Turkey	5	596	576	97
United Kingdom	3	650	590	91
United States	1	440	435	99
United States	40	440	438	100
