# Supplementary material for: The Twitter parliamentarian database: Analyzing Twitter politics across 26 countries
Source: PLoS One. 2020 Sep 16;15(9):e0237073. doi: 10.1371/journal.pone.0237073 (PMC7494116; doi:10.1371/journal.pone.0237073)
Supplement: S2 Table — (PDF) [file pone.0237073.s004.pdf]

S2 Table. This table shows clustering and degree measures, as well as fractions of external mentions and the strength of relationship between party and cluster membership, applied to the individual country networks.

Country	Democratic system	Type	Fraction external retweets	Average modularity	Clusters (N)	Average clustering coefficient	Degree kurtosis	Cramer 's V
Australia	M	2	0.04	0.575	8	0.399	-0.24	0.679
Belgium	PR	1	0.23	0.793	7	0.398	2.37	0.929
Canada	M	1	0.08	0.550	9	0.451	3.12	0.857
Denmark	PR	4	0.16	0.583	6	0.326	0.60	0.793
Finland	PR	3	0.20	0.560	7	0.342	-0.66	0.696
Germany	Mixed	1, 3	0.19	0.716	9	0.352	0.32	0.896
Ireland	PR	2	0.04	0.529	5	0.5	-0.80	0.950
Italy	Mixed	1	0.26	0.686	6	0.348	4.73	0.767
Malta	PR	2	0.05	0.461	5	0.594	-0.56	1.000
Netherlands	PR	3	0.13	0.656	7	0.396	-0.05	0.859
New Zealand	Mixed	2	0.08	0.562	6	0.402	0.68	0.650
Norway	PR	4	0.21	0.623	8	0.212	-0.56	0.612
Poland	PR	2	0.04	0.518	5	0.47	0.57	0.699
Spain	PR	1	0.11	0.677	9	0.471	0.68	0.739
Sweden	PR	2	0.33	0.687	9	0.277	1.43	0.826
Switzerland	PR	4	0.16	0.520	8	0.331	1.24	0.577
Turkey	PR	1	0.10	0.651	10	0.274	2.28	0.926
United Kingdom	M	2	0.08	0.519	9	0.328	1.03	0.590
United States	M	2	0.14	0.517	14	0.281	8.26	0.813
