# Supplementary material for: The Twitter parliamentarian database: Analyzing Twitter politics across 26 countries
Source: PLoS One. 2020 Sep 16;15(9):e0237073. doi: 10.1371/journal.pone.0237073 (PMC7494116; doi:10.1371/journal.pone.0237073)
Supplement: S3 Table — High values indicate that a term occurs more frequently than chance amongst one party. (PDF) [file pone.0237073.s005.pdf]

S3 Table. This table shows the highest Log-Likelihood values (shown in brackets) of hashtags used by the two largest parties in 2018. High values indicate that a term occurs more frequently than chance amongst one party.

**Australia**	
Labour	*The Nationals*
auspol (634)	lovegippsland (1608)
politas (480)	getactive (1345)
qt (316)	regionsmatter (1310)
youradf (111)	lovesport (1198)
ausdef (99)	parkeselectorate (972)
insiders (96)	tyfys (646)
estimates (83)	ruralhealth (364)
lestweforget (73)	improud (326)
ausvotes (65)	riverina (318)
nbn (63)	wombattrail (289)
**Canada**	
Labour	*Conservative*
barrie (4158)	cdnpoli (3996)
innisfil (4103)	polcan (591)
cpc (1896)	scarbto (519)
onpoli (1398)	yourbudget2019 (397)
eml (1330)	lib2018 (347)
lavscam (1167)	goc (315)
yxe (1156)	ottawacentre (314)
cpc_hq (1055)	yourbudget2018 (311)
kitcon (921)	parkhp (308)
skpoli (846)	hamont (299)
**United Kingdom**	
Labour	*Conservative*
brexit (13873)	harlow (4868)
pmqs (6637)	torbayhour (4825)
peoplesvote (4610)	cpc17 (4409)
labourdoorstep (4562)	boosttorbay (4390)
tomorrowspaperstoday (4084)	ukaid (3912)
plymouth (3471)	torycanvass (3725)
lab17 (2855)	cpc18 (3372)
brexitshambles (2728)	southend (3163)
forthemany (2254)	crawley (2687)
universalcredit (2248)	backthebrexitdeal (2618)
**United States**	
Republican	*Democrat*
taxreform (12248)	goptaxscam (5464)
utpol (5086)	trumpcare (3961)
taxcutsandjobsact (4631)	trumpshutdown (3572)
ohio (2656)	netneutrality (2825)
betteroffnow (2429)	protectourcare (2566)
va10 (2090)	forthepeople (2363)
venezuela (1935)	getcovered (1879)
sofla (1783)	aca (1650)
schumershutdown (1760)	dreamers (1357)
ms01 (1410)	protectdreamers (1352)
