# Supplementary material for: The Twitter parliamentarian database: Analyzing Twitter politics across 26 countries
Source: PLoS One. 2020 Sep 16;15(9):e0237073. doi: 10.1371/journal.pone.0237073 (PMC7494116; doi:10.1371/journal.pone.0237073)
Supplement: S4 Table — (PDF) [file pone.0237073.s006.pdf]

S4 Table. This table shows the number and percentage of MPs who mentioned another politician in the EFTA in 2018.

Country	MPs that made at least one external mention (N)	MPs that made at least one external mention (%)
Austria	24	37
Belgium	41	32
Denmark	64	40
Finland	55	32
France	271	53
Germany	195	38
Greece	28	27
Iceland	12	30
Ireland	102	68
Italy	60	13
Latvia	14	34
Malta	31	53
Netherlands	78	53
Norway	30	26
Poland	95	28
Spain	126	58
Sweden	57	35
Switzerland	38	31
United Kingdom	318	54
